# Awareness of Medico-Legal Case Management Among Saudi Board Emergency Medicine Physicians in Riyadh: A Multicenter Cross-Sectional Study

**DOI:** 10.7759/cureus.113948

**Published:** 2026-08-04

**Authors:** Dohayan A Aldohayan, Bader Alghamdi, Rayan S Molawi, Rayan Alfaleh

**Affiliations:** 1 Department of Emergency Medicine, Prince Sultan Military Medical City, Riyadh, SAU

**Keywords:** emergency medicine, emergency physicians, evidence preservation, forensic documentation, medical law, medico-legal cases, saudi arabia

## Abstract

Background: Emergency physicians frequently manage cases with legal implications. Accurate documentation, evidence preservation, and compliance with reporting requirements are essential, yet awareness of these duties may be incomplete.

Objective: To describe awareness of medico-legal case management among Saudi Board Emergency Medicine physicians in Riyadh and explore associations with demographic and professional characteristics.

Methods: This multicenter cross-sectional study included emergency medicine residents, specialists/registrars, and consultants from seven governmental and tertiary hospitals in Riyadh. A 10-item investigator-developed awareness questionnaire and five self-reported confidence items were administered electronically. The instrument was reviewed by five consultant emergency physicians for clarity, relevance, comprehensiveness, and clinical applicability; no pilot study, formal content validity index, Delphi process, or construct validity assessment was performed. For analysis, a "Yes" response to each awareness item was coded 1 and a "No" or "Don't know" response was coded 0, yielding a 0-10 index. Investigator-defined cutoffs of 0-3, 4-7, and 8-10 were used to label poor, moderate, and good awareness, respectively; these cutoffs have not been externally validated. Descriptive statistics, univariable tests, and multivariable linear regression with heteroscedasticity-robust standard errors were used.

Results: Of 576 submitted questionnaires, 276 incomplete submissions were excluded, and 300 complete responses were analyzed. The mean awareness index was 6.31 ± 1.59 (median, 6; observed range, 1-10). Correct responses varied across the 10 awareness items, ranging from 56.3% to 71.3%, with the lowest proportions observed for photographic documentation and legal consequences of failing to report suspected medico-legal cases, and the highest for workplace evidence-collection protocols. Internal consistency was low (Cronbach’s alpha = 0.097), indicating that the awareness index should be interpreted as an exploratory summary of heterogeneous medico-legal domains. In the adjusted model, compared with consultants, R4, R3, and R2 residents had lower awareness scores, while managing two to five medico-legal cases per shift was associated with a slightly higher score than managing fewer than two cases. Previous report-writing training was not independently associated with awareness.

Conclusion: Physicians demonstrated variable awareness across different medico-legal knowledge domains. As the investigator-developed awareness index demonstrated very low internal consistency, the overall score should be interpreted cautiously as an exploratory summary rather than a validated measure of a single construct. Future studies should prospectively validate a multidimensional instrument and evaluate competency-based medico-legal education.

## Introduction

Emergency departments (EDs) are the frontline of healthcare systems and frequently provide the initial assessment and management of patients involved in trauma, interpersonal violence, poisoning, child abuse, road traffic injuries, occupational accidents, and unexplained deaths. Many of these presentations constitute medico-legal cases (MLCs), requiring emergency physicians to fulfill responsibilities that extend beyond clinical management to include legal, ethical, and professional obligations. Appropriate MLC management requires timely identification of MLCs, accurate documentation, preservation of forensic evidence, maintenance of the chain of custody, and prompt reporting to the appropriate legal authorities. Failure to perform these responsibilities appropriately may compromise forensic investigations, delay judicial proceedings, violate patients’ legal rights, and expose physicians and healthcare institutions to medico-legal liability [1-5].

Effective management of MLCs requires not only sound clinical competence but also adequate knowledge of medico-legal principles, institutional policies, and national regulations. Emergency physicians are expected to recognize cases requiring mandatory reporting, prepare comprehensive medico-legal reports, preserve forensic evidence appropriately, and comply with legal and ethical standards governing healthcare practice. These responsibilities are commonly performed in high-pressure emergency settings where rapid decision-making is essential. Consequently, insufficient medico-legal knowledge or uncertainty regarding institutional procedures may result in incomplete documentation, improper evidence handling, delayed reporting, and increased medico-legal risk [1-5].

Previous studies conducted in Saudi Arabia and internationally have reported deficiencies in physicians’ medico-legal knowledge, documentation practices, and formal medico-legal education despite generally acceptable theoretical awareness. Studies performed among emergency physicians in the Eastern Province and Jeddah demonstrated moderate overall awareness while identifying important deficiencies in medico-legal documentation, evidence preservation, and report-writing practices. Similar findings have also been reported internationally, emphasizing the ongoing need for structured medico-legal education and standardized institutional training programs [3,6-10].

In Saudi Arabia, increasing public awareness of patients’ rights, expansion of emergency medical services, and the growing number of medical malpractice claims have further emphasized physicians’ medico-legal responsibilities. National regulations and professional guidance issued by the Saudi Commission for Health Specialties require healthcare practitioners to maintain accurate documentation, preserve confidentiality, comply with mandatory reporting requirements, and practice according to established ethical and professional standards [1,5,11,12].

Despite these developments, evidence regarding medico-legal awareness among emergency physicians practicing in Riyadh remains limited. Most previous Saudi studies have been conducted in the Eastern Province or Jeddah, whereas Riyadh represents the largest healthcare and postgraduate emergency medicine training center in the Kingdom. Evaluating physicians’ awareness in this setting is essential for identifying existing knowledge gaps and supporting future improvements in emergency medicine education and institutional policies.

Therefore, this multicenter cross-sectional study aimed to assess awareness of MLC management among Saudi Board Emergency Medicine physicians practicing in Riyadh and to examine its association with selected demographic and professional characteristics, including gender, professional designation, emergency medicine experience, previous medico-legal education, previous report-writing training, and MLC workload.

## Materials and methods

Study design and setting

A multicenter cross-sectional study was conducted among Saudi Board Emergency Medicine physicians working in seven governmental and tertiary hospitals in Riyadh, Saudi Arabia.

Participants and sampling

Eligible participants were residents (R1-R4), specialists/registrars, and consultants actively practicing emergency medicine in participating hospitals and providing electronic informed consent. Physicians from other specialties, physicians practicing outside Riyadh, and incomplete submissions were excluded. Convenience sampling was used, and the questionnaire link was distributed through professional communication networks. As the number of eligible physicians who received the invitation was not recorded, a true response rate could not be calculated.

Sample size

No a priori sample size calculation was performed because the study used convenience sampling during the predefined study period. Consequently, all inferential analyses should be interpreted as exploratory. A retrospective precision estimate indicates that a sample of 300 corresponds to a maximum absolute precision of approximately 5.7% at the 95% confidence level under simple random-sampling assumptions. This estimate is provided for context only and does not replace an a priori sample size calculation.

Questionnaire development and scoring

The questionnaire was independently developed by the investigators after reviewing literature on MLC management. Five consultant emergency physicians reviewed the preliminary instrument for clarity, relevance, comprehensiveness, and clinical applicability, after which wording and sequence were refined. No pilot study, formal content validity index, Delphi process, or construct-validity assessment was conducted.

The questionnaire contained demographic and professional items, 10 awareness items, and five self-reported confidence statements. For analysis, a "Yes" response to each awareness item was coded 1 and a "No" or "Don't know" response was coded 0. Total index scores ranged from 0 to 10. For descriptive purposes only, investigator-defined cutoffs (0-3, 4-7, and 8-10) were explored to summarize the distribution of awareness scores. These categories were not externally validated and were not used as the primary basis for interpretation. Confidence items used a five-point Likert response format and were reported item by item. As the confidence items did not demonstrate acceptable internal consistency, no composite confidence scale was used for inferential analysis.

Data collection and participant flow

**Figure 1:**
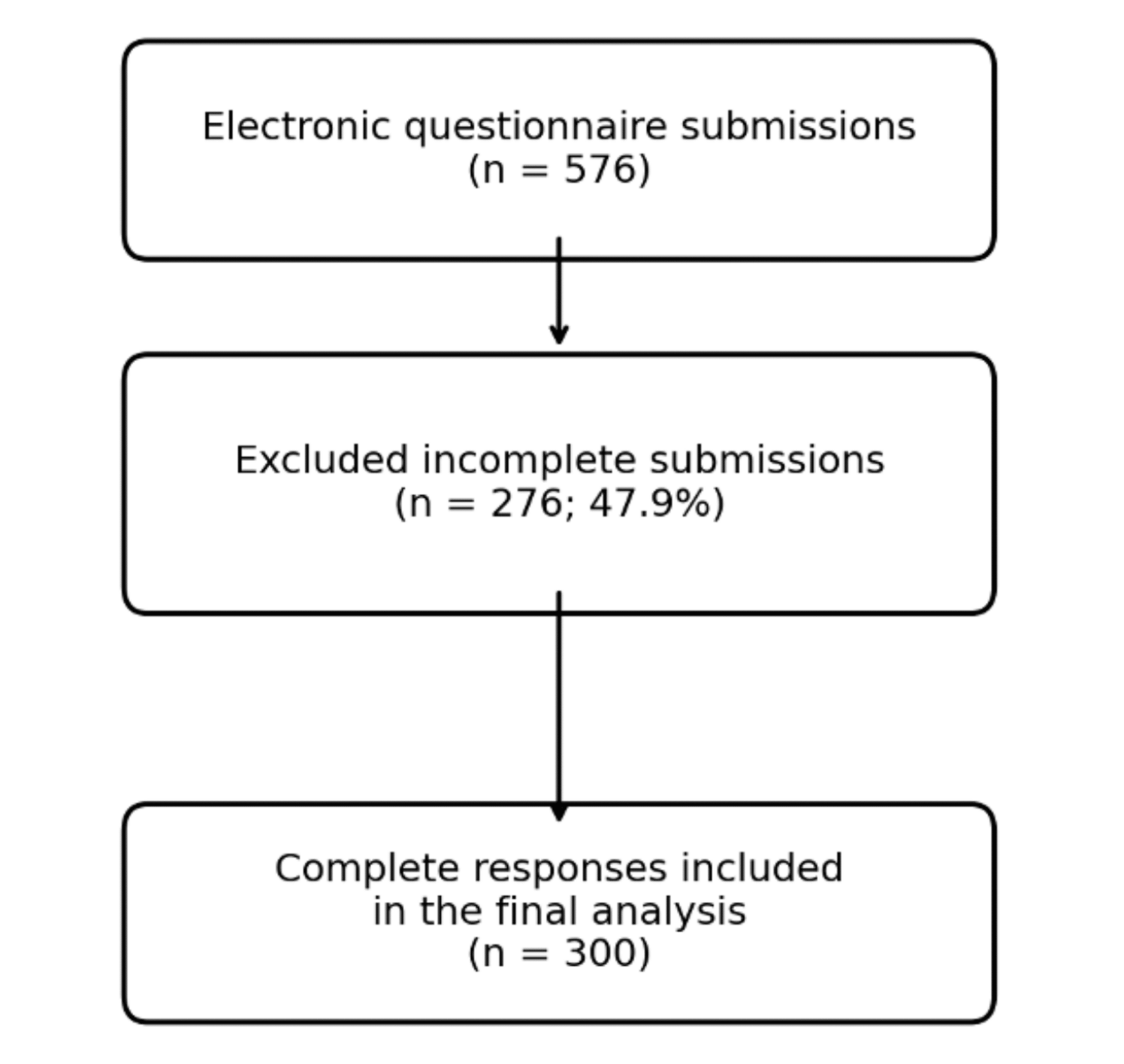
Participant flow through data cleaning and analysis.

The questionnaire was administered anonymously through Google Forms (Google, Mountain View, CA). The dataset contained 576 submitted records. During cleaning, 276 records lacked complete required fields and were excluded; 300 complete records were included. The 52.1% figure represents the proportion of submitted records that were complete, not a response rate (Figure 1).

Statistical analysis

Analyses were performed using the complete-case dataset. Categorical variables are summarized as frequencies and percentages. Awareness score is summarized using mean, standard deviation, median, minimum, and maximum. Internal consistency was assessed with Cronbach's alpha. Distributional shape was examined using a histogram, Q-Q plot, skewness, kurtosis, and the Shapiro-Wilk test. As the awareness index was approximately symmetric but discrete, multivariable linear regression with HC3 robust standard errors was used to estimate associations while reducing sensitivity to violations of homoscedasticity assumptions. Predictors were gender, professional designation, emergency medicine experience, prior medico-legal education, prior report-writing training, and average MLCs managed per shift. Regression coefficients are reported with 95% confidence intervals and p-values. Two-sided p < 0.05 was considered statistically significant. No post hoc pairwise comparisons were conducted after the non-significant univariable ANOVA for designation.

Ethical considerations

Ethical approval was obtained from the Prince Sultan Military Medical City Scientific Research Center Institutional Review Board (Approval No. E-2956; approval date: 13 July 2026) before study commencement. Electronic informed consent was obtained, participation was voluntary, and responses were anonymized. The study was conducted in accordance with the Declaration of Helsinki.

## Results

Participant characteristics

Among 300 participants analyzed, 206 (68.7%) were male, and 94 (31.3%) were female. All residency levels, specialists/registrars, and consultants were represented. Table 1 summarizes participant characteristics.

**Table 1:** Demographic and professional characteristics of the participants (N = 300).

Characteristic	Category	n (%)
Gender	Male	206 (68.7)
	Female	94 (31.3)
Professional designation	Consultant	26 (8.7)
	Specialist/Registrar	49 (16.3)
	R4 Resident	66 (22.0)
	R3 Resident	81 (27.0)
	R2 Resident	53 (17.7)
	R1 Resident	25 (8.3)
Experience	<5 years	128 (42.7)
	5-9 years	98 (32.7)
	≥10 years	74 (24.7)
Previous medico-legal education	None	90 (30.0)
	Undergraduate	102 (34.0)
	Postgraduate	60 (20.0)
	Short course/workshop	48 (16.0)
Previous report-writing training	Yes	112 (37.3)
	No	188 (62.7)
Cases managed per shift	<2	105 (35.0)
	2-5	134 (44.7)
	>5	61 (20.3)

Awareness score and item responses

The 0-10 awareness index had a mean of 6.31 (SD, 1.59), a median of 6, and an observed range of 1-10. The Shapiro-Wilk test was significant (W = 0.959, p < 0.001), although skewness (-0.19) and excess kurtosis (-0.05) were small. Cronbach's alpha was 0.097, indicating very low internal consistency. The total score should therefore be interpreted cautiously as an exploratory summary across heterogeneous medico-legal topics rather than as a validated unidimensional scale (Table 2).

**Table 2:** Item-level awareness responses (N = 300). Correct responses ranged from 56.3% to 71.3% across the 10 awareness items, demonstrating variability in physicians’ knowledge across different medico-legal domains. The lowest proportions of correct responses were observed for photographic documentation (56.3%) and the legal consequences of failing to report suspected medico-legal cases (57.3%), whereas the highest proportion was observed for awareness of workplace evidence-collection protocols (71.3%). The mean awareness index was 6.31 ± 1.59 (median, 6; observed range, 1-10). Cronbach’s alpha was 0.097, indicating that the index should be interpreted cautiously as an exploratory summary rather than a validated unidimensional scale.

Awareness item	Yes response, n (%)	No/Don't know, n (%)
Police notification is legally required in suspected criminal cases	190 (63.3)	110 (36.7)
All dead-on-arrival cases should be evaluated for medico-legal suspicion	203 (67.7)	97 (32.3)
Police notification should precede disclosure to relatives in suspicious cases	190 (63.3)	110 (36.7)
Failure to report a suspected medico-legal case may have legal consequences	172 (57.3)	128 (42.7)
Emergency physicians are legally responsible for proper medico-legal documentation	185 (61.7)	115 (38.3)
Hospital has a unified protocol for medico-legal case management	203 (67.7)	97 (32.3)
Standard guidelines are available for completing medico-legal reports	184 (61.3)	116 (38.7)
Photographic documentation is recommended in selected medico-legal cases	169 (56.3)	131 (43.7)
Preservation of clothing and biological samples is important	184 (61.3)	116 (38.7)
Workplace provides a protocol for evidence collection	214 (71.3)	86 (28.7)

Self-reported confidence

Findings related to the participants' self-reported confidence in MLC management are presented in Table 3.

**Table 3:** Self-reported confidence in medico-legal case management (N = 300). The five confidence items had negative internal consistency (Cronbach's alpha = -0.481), indicating that they should not be combined into a single composite score. They were therefore interpreted item by item.

Statement	Strongly disagree	Disagree	Neutral	Agree	Strongly agree
Confident in identifying medico-legal cases	13 (4.3)	44 (14.7)	80 (26.7)	91 (30.3)	72 (24.0)
Confident in completing medico-legal reports correctly	0 (0.0)	34 (11.3)	70 (23.3)	121 (40.3)	75 (25.0)
Routinely follows proper evidence-preservation procedures	0 (0.0)	62 (20.7)	88 (29.3)	78 (26.0)	72 (24.0)
Feels adequately trained to manage medico-legal cases	0 (0.0)	27 (9.0)	76 (25.3)	127 (42.3)	70 (23.3)
Incomplete medico-legal reports reduce legal value	0 (0.0)	38 (12.7)	66 (22.0)	123 (41.0)	73 (24.3)

Univariable and multivariable analyses

In univariable analyses, awareness score did not differ significantly by gender, designation, experience, report-writing training, or workload. Scores differed by prior medico-legal education (ANOVA p = 0.017), with the short-course/workshop group having the lowest mean. The designation ANOVA was not significant (p = 0.064), so no post hoc tests were performed (Tables 4, 5).

**Table 4:** Univariable comparisons of awareness scores. The adjusted linear model was statistically significant overall (robust F = 2.13, p = 0.011), with R² = 0.103 and adjusted R² = 0.059. Compared with consultants, R4, R3, and R2 residents had lower adjusted mean awareness scores. Participants managing two to five cases per shift had a modestly higher adjusted mean score than those managing fewer than two cases. Previous report-writing training was not independently associated with awareness.

Variable	Category	n	Mean ± SD	95% CI	p-value
Gender	Male	206	6.24 ± 1.57	6.02-6.45	0.234
	Female	94	6.48 ± 1.64	6.14-6.82	
Designation	Consultant	26	7.08 ± 1.32	6.54-7.61	0.064
	Specialist/Registrar	49	6.61 ± 1.55	6.17-7.06	
	R4	66	6.15 ± 1.71	5.73-6.57	
	R3	81	6.07 ± 1.50	5.74-6.41	
	R2	53	6.23 ± 1.59	5.79-6.66	
	R1	25	6.32 ± 1.73	5.61-7.03	
Experience	<5 years	128	6.37 ± 1.56	6.09-6.64	0.107
	5-9 years	98	6.49 ± 1.61	6.17-6.81	
	≥10 years	74	5.99 ± 1.59	5.62-6.36	
Education	None	90	6.54 ± 1.55	6.22-6.87	0.017
	Undergraduate	102	6.38 ± 1.52	6.08-6.68	
	Postgraduate	60	6.37 ± 1.57	5.96-6.77	
	Short course/workshop	48	5.67 ± 1.72	5.17-6.17	
Report-writing training	Yes	112	6.21 ± 1.60	5.92-6.51	0.407
	No	188	6.37 ± 1.59	6.14-6.60	
Cases/shift	<2	105	6.06 ± 1.65	5.74-6.38	0.095
	2-5	134	6.51 ± 1.58	6.24-6.78	
	>5	61	6.33 ± 1.49	5.95-6.71	

**Table 5:** Multivariable linear regression of awareness score with HC3 robust standard errors.

Gender: Female vs. Male	0.18	-0.23 to 0.59	0.387
Designation: Specialist/Registrar vs. Consultant	-0.44	-1.17 to 0.29	0.235
Designation: R4 vs. Consultant	-0.91	-1.61 to -0.21	0.010
Designation: R3 vs. Consultant	-0.94	-1.58 to -0.30	0.004
Designation: R2 vs. Consultant	-0.86	-1.59 to -0.13	0.021
Designation: R1 vs. Consultant	-0.73	-1.60 to 0.15	0.103
Experience: 5-9 years vs. <5 years	0.16	-0.28 to 0.61	0.469
Experience: ≥10 years vs. <5 years	-0.32	-0.78 to 0.15	0.180
Education: Undergraduate vs. None	-0.14	-0.60 to 0.31	0.541
Education: Postgraduate vs. None	-0.23	-0.78 to 0.32	0.417
Education: Short course/workshop vs. None	-0.88	-1.49 to -0.28	0.004
Report training: Yes vs. No	-0.23	-0.63 to 0.16	0.250
Cases/shift: 2-5 vs. <2	0.43	0.01 to 0.86	0.044
Cases/shift: >5 vs. <2	0.22	-0.29 to 0.73	0.398

## Discussion

This multicenter study found that awareness of MLC management varied across individual knowledge domains. The lowest awareness was observed for photographic documentation and the legal consequences of failing to report suspected MLCs, whereas awareness was highest for workplace evidence-collection protocols. The low internal consistency likely reflects that the questionnaire assessed multiple distinct domains of medico-legal knowledge rather than a single latent construct. Accordingly, the awareness index should be interpreted as an exploratory summary of heterogeneous medico-legal topics rather than a validated measure of a single construct.

Moderate performance despite previous educational exposure may reflect deficiencies in how medico-legal content is delivered. Training may be episodic, lecture-based, and focused on legal concepts rather than repeated practice in documentation, chain-of-custody procedures, evidence handling, and reporting decisions. Curricula may also vary among training sites, with limited use of simulation, case-based assessment, direct observation, and feedback. In high-pressure emergency settings, knowledge acquired in isolated sessions may not transfer reliably to practice. These explanations are plausible but were not directly tested in this cross-sectional study.

The absence of an independent association between previous report-writing training and awareness should not be interpreted as evidence that training is ineffective. The study recorded only whether training had occurred, without measuring its duration, recency, content, quality, or assessment strategy. Misclassification of educational exposure, the low reliability of the awareness index, and residual confounding may all attenuate associations.

Adjusted differences by professional designation and workload were small and should be interpreted cautiously. The regression model explained only about 10% of score variation, and the cross-sectional design cannot establish temporal or causal relationships. These findings are hypothesis-generating rather than evidence that designation or workload determines awareness.

The results are broadly consistent with prior Saudi and international studies describing gaps in medico-legal documentation, evidence preservation, and training [3,6-10]. Nevertheless, direct numerical comparisons are limited because instruments, scoring approaches, legal frameworks, and study populations differ. Future work should first develop and validate a multidimensional instrument, then evaluate competency-based interventions prospectively.

Strengths and limitations

Strengths include recruitment across seven hospitals, a sample of 300 complete responses, inclusion of all residency levels together with specialists/registrars and consultants, and analysis of multiple professional variables in an adjusted model.

Several limitations substantially qualify the findings. First, the questionnaire was investigator-developed and, although reviewed by five emergency medicine consultants, was not piloted and did not undergo formal content validity index, Delphi, construct-validity, test-retest, or other psychometric evaluation. The awareness index had very low internal consistency, and the confidence items had negative internal consistency. Second, no a priori sample size calculation was performed. Third, convenience sampling and electronic distribution may have preferentially attracted physicians interested in medico-legal issues. Fourth, 276 of 576 submissions (47.9%) were incomplete and excluded; as characteristics of incomplete respondents and the number invited were unavailable, selection and non-response bias could not be assessed. Fifth, self-report may be affected by recall and social desirability bias and does not establish actual clinical performance. Sixth, the study was limited to Riyadh and may not generalize nationally. Finally, the cross-sectional design precludes causal inference, and some potentially relevant variables, including age, were not available in the analyzed dataset.

## Conclusions

Physicians demonstrated variable awareness across different medico-legal knowledge domains, with the greatest deficiencies observed in photographic documentation and legal reporting responsibilities. As the investigator-developed awareness index demonstrated very low internal consistency, the overall score should be interpreted cautiously as an exploratory summary rather than a validated measure of a single construct. Future studies should develop and prospectively validate a multidimensional instrument before evaluating standardized competency-based educational interventions and institutional protocols.

## Appendices

Appendix 1. Complete questionnaire and scoring

Section I: Demographic and Professional Characteristics

**Table 6:** Demographic and professional questionnaire items.

Question	Response options
Gender	Male; Female
Professional designation	R1; R2; R3; R4; Specialist/Registrar; Consultant
Emergency medicine experience	<5 years; 5-9 years; ≥10 years
Previous medico-legal or forensic education	None; Undergraduate; Postgraduate; Short course/workshop
Previous medico-legal report-writing training	Yes; No
Average medico-legal cases per shift	<2; 2-5; >5

Section II: Awareness Items

**Table 7:** Awareness questionnaire items. Exploratory descriptive categories used only for presentation: Poor (0–3), Moderate (4–7), and Good (8–10). These cutoffs were investigator-defined and have not been externally validated.

No.	Item	Response options
1	Police notification is legally required in suspected criminal cases	Yes; No; Don't know
2	All dead-on-arrival cases should be evaluated for medico-legal suspicion	Yes; No; Don't know
3	Police notification should precede disclosure to relatives in suspicious cases	Yes; No; Don't know
4	Failure to report a suspected medico-legal case may have legal consequences	Yes; No; Don't know
5	Emergency physicians are legally responsible for proper medico-legal documentation	Yes; No; Don't know
6	Hospital has a unified protocol for medico-legal case management	Yes; No; Don't know
7	Standard guidelines are available for completing medico-legal reports	Yes; No; Don't know
8	Photographic documentation is recommended in selected medico-legal cases	Yes; No; Don't know
9	Preservation of clothing and biological samples is important	Yes; No; Don't know
10	Workplace provides a protocol for evidence collection	Yes; No; Don't know

Section III: Self-Reported Confidence

**Table 8:** Self-reported confidence items.

No.	Statement	Response options
1	Confident in identifying medico-legal cases	Strongly disagree; Disagree; Neutral; Agree; Strongly agree
2	Confident in completing medico-legal reports correctly	Strongly disagree; Disagree; Neutral; Agree; Strongly agree
3	Routinely follows proper evidence-preservation procedures	Strongly disagree; Disagree; Neutral; Agree; Strongly agree
4	Feels adequately trained to manage medico-legal cases	Strongly disagree; Disagree; Neutral; Agree; Strongly agree
5	Incomplete medico-legal reports reduce legal value	Strongly disagree; Disagree; Neutral; Agree; Strongly agree

## References

[1] Ministry of Health, Saudi Arabia (2021). Ministry of Health, Saudi Arabia. Law of practicing healthcare professions and its executive regulations. Ministry of Health.

[2] World Health Organization (2003). Guidelines for Medico-Legal Care for Victims of Sexual Violence. https://iris.who.int/server/api/core/bitstreams/67468e99-5b64-40d9-b68b-c0e55923f32e/content.

[3] Madadin M, Alqarzaie AA, Alzahrani RS, Alzahrani FF, Alqarzea SM, Alhajri KM, Al Jumaan MA (2021). Characteristics of medico-legal cases and errors in medico-legal reports at a teaching hospital in Saudi Arabia. Open Access Emerg Med.

[4] Smith JD, Lemay K, Lee S, Nuth J, Ji J, Montague K, Garber GE (2023). Medico-legal issues related to emergency physicians' documentation in Canadian emergency departments. CJEM.

[5] Saudi Commission for Health Specialties (2014). Saudi Commission for Health Specialties. Code of ethics for healthcare practitioners. Riyadh: Saudi Commission for Health Specialties.

[6] Alabdulqader S, Alabdulqader R, Madadin M, Kashif H, Al Jumaan MA, Yousef AA, Menezes RG (2023). Emergency physicians' awareness of medico-legal case management: a cross-sectional study from Saudi Arabia. Saudi J Med Med Sci.

[7] Zaki MK, Bayoumi KA, Rawas M, Alshutayri ZN, Kensarah EA, Jamal AM, Zughbi MA (2018). Measuring the awareness of emergency department physicians toward the management of medicolegal cases in Jeddah, Kingdom of Saudi Arabia. Saudi J Forensic Med Sci.

[8] Shihah AS, Alrashed AH, Al-Abduljabbar KA, Alamri HA, Shafiq M, Ahmed MN, Alkhenizan AH (2022). Awareness of medical law among health care practitioners in Saudi Arabia. Saudi Med J.

[9] Mokhtar M, Azab SM, Hassan S, Ez-Elarab HS (2018). Study of handling of medico-legal cases in governmental hospitals in Cairo. J Forensic Leg Med.

[10] Gurpur NN, Saldanha S, Shet N, Sharma P (2019). Challenges faced in handling the medico-legal cases in a selected teaching hospital. Int J Community Med Public Health.

[11] Alkhenizan AH, Shafiq MR (2018). The process of litigation for medical errors in Saudi Arabia and the United Kingdom. Saudi Med J.

[12] Samarkandi A (2006). Status of medical liability claims in Saudi Arabia. Ann Saudi Med.

